# Thyroid abscess, an uncommon diagnosis: A case report and mini‑review of the literature

**DOI:** 10.3892/mi.2024.153

**Published:** 2024-04-04

**Authors:** Patricia Urbón-sánchez, Fernando Mendoza-Moreno, Sofía Sánchez De Toca-Gómez, Félix Mañes-Jiménez, Rubén Jiménez-Martín, Marta Bru-Aparicio, Pilar Laguna-Hernández, Yousef Allaoua-Moussaoui, Sonia Soto-Schutte, Ana Quiroga-Valcárcel, Manuel Díez-Alonso, Inmaculada Lasa Unzúe, Alberto Gutiérrez-Calvo

**Affiliations:** Department of General and Digestive Surgery, Príncipe de Asturias Teaching Hospital, 28805 Madrid, Spain

**Keywords:** thyroid, abscess, emergency, antibiotics, hemithyroidectomy

## Abstract

Thyroid abscess is a rare entity, commonly experienced by immunocompromised patients, or those who have anatomical abnormalities or a pre-existing thyroid disease. An early diagnosis continued by treatment with antibiotics and drainage of the abscess is the recommended therapeutic strategy for such cases. The present study describes a clinical case of this rare event, and also provides a brief literature review. The present study describes the case of a 48-year-old healthy male with no medical antecedents, apart from acute prostatitis treated with antibiotics for 6 days prior, who visited the Emergency Department of the authors' hospital with neck pain and progressive swelling of the mass. Diagnostic imaging confirmed the authors' suspicion of an abscess and revealed the lesion displacing the airway to the contralateral side. This restricted the mobility of the neck of the patient. As an emergency measure, the patient was then taken to the operating room for a neck examination. A hemithyroidectomy was finally performed. Following a prolonged hospital duration, he was discharged from the hospital and his recovery was uneventful without any voice alterations, hypocalcemia or recurrence.

## Introduction

Thyroid abscess is a rare entity, comprising 0.1-0.7% of all thyroid diseases (1) due to the natural immunity to infection of the thyroid gland, owing to its anatomical and physiological characteristics (2). However, when an infection does occur, it can lead to the formation of an abscess. The majority of presentations occur in patients who have anatomic defects, pre-existing thyroid disease or compromised immune systems. Diagnosis typically involves a combination of clinical findings and diagnostic imaging, with ultrasound being the initial investigation. Treatment of thyroid abscesses includes antimicrobial therapy with drainage or thyroidectomy. It is critical for an early diagnosis to be made and for treatment to be administered immediately, as the condition can lead to severe morbidity if left untreated (3).

The present study describes the case of a patient who presented to the Emergency Department of Príncipe de Asturias Teaching Hospital (Alcalá de Henares, Madrid, Spain) presenting cervical neck pain due to a thyroid abscess without any risk factors and no previous history of thyroid diseases. In addition, the present study provides a brief review of the literature, with the aim of providing an overview of the disease, as well as the presentation and management of this entity.

## Case report

A 48-year-old male with no previous medical history apart from acute prostatitis (*Escherichia coli* identified in urine cultures obtained from the patient) treated with ciprofloxacin for 6 days presented to the Emergency Department of Príncipe de Asturias Teaching Hospital (Alcalá de Henares, Madrid, Spain) with a 3-day history of a painful right neck and swelling. The pain was constant, radiating to the right ear, and was accompanied by difficulty to swallow and odynophagia. The patient also complained of an ipsilateral headache and of restricted mobility of the neck. He denied having any fever or dysthymia.

**Figure 1:**
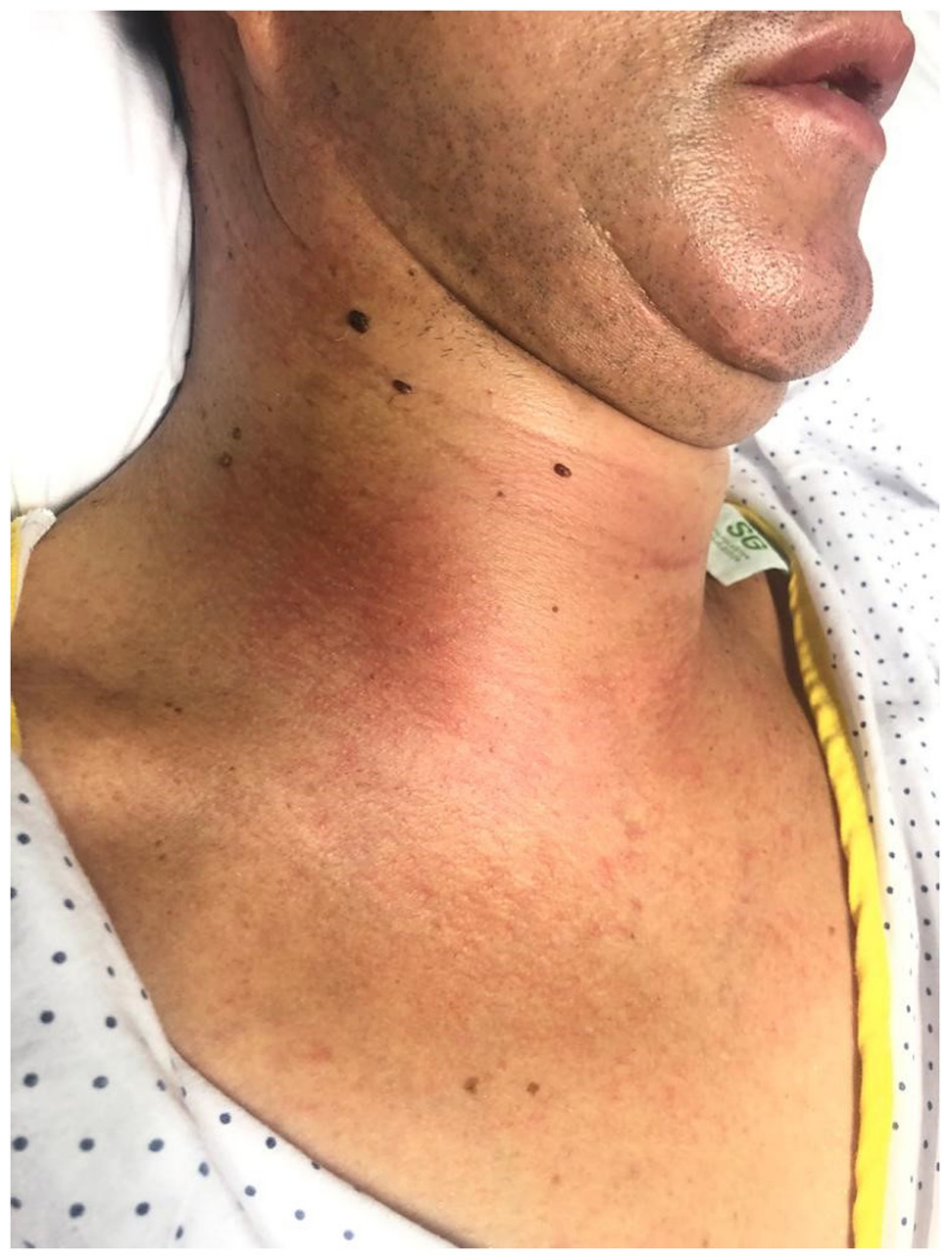
The patient described herein presented with cervical right neck swelling.

A physical examination revealed an indurated, warm and painful mass (5 cm in size) in the anterolateral right neck (Fig. 1). The other side of the neck appeared normal. The remainder of the systemic examination did not reveal any notable results. During his stay in the emergency room, he was afebrile and hemodynamically stable.

Laboratory tests revealed a white blood cell count of 16.100 µl with a neutrophil count of 12.700 µl, hemoglobin levels of 9.7 mg/dl, a platelet count of 728.000 µl and C reactive protein levels of 147.6 mg/l.

An emergency cervical neck ultrasound was requested, identifying a rounded lesion measuring 6.6x6.3x5.4 cm that appeared to be arising from the right thyroid lobe, with well-defined borders, a thick wall and echogenic content within, as well as some septation and peripheral vascularity, suggesting a complicated cyst with bleeding/infection.

**Figure 2:**
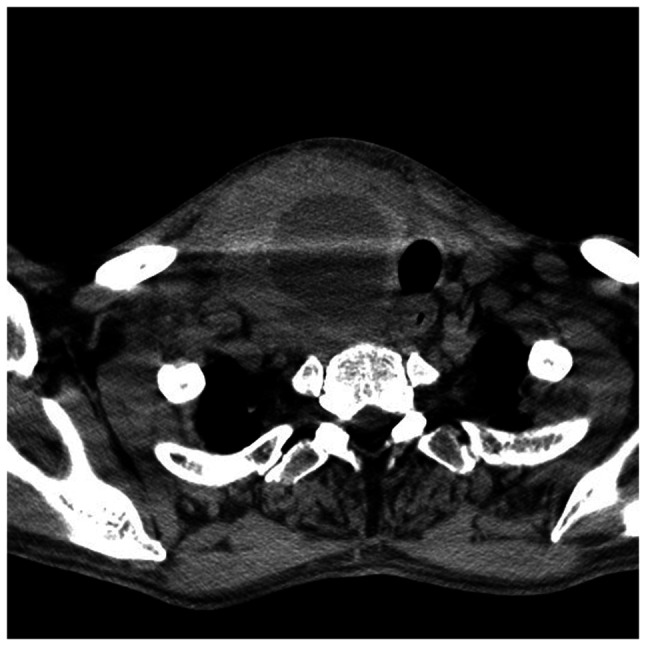
Computed tomography scan illustrating the lesion displacing the trachea and narrowing the caliber of the jugular vein in different cross-sections to show its extension.

Subsequently, a computed tomography (CT) scan of the neck also revealed a heterogeneous cystic mass with thickened enhancing walls occupying the entire right lateral cervical space, displacing the airway to the contralateral side, compressing the right thyroid lobe, and displacing and narrowing the caliber of the jugular vein. This provided conclusive evidence of a right lateral cervical abscess and infection affecting all the fat spaces in the neck (Fig. 2).

**Figure 3:**
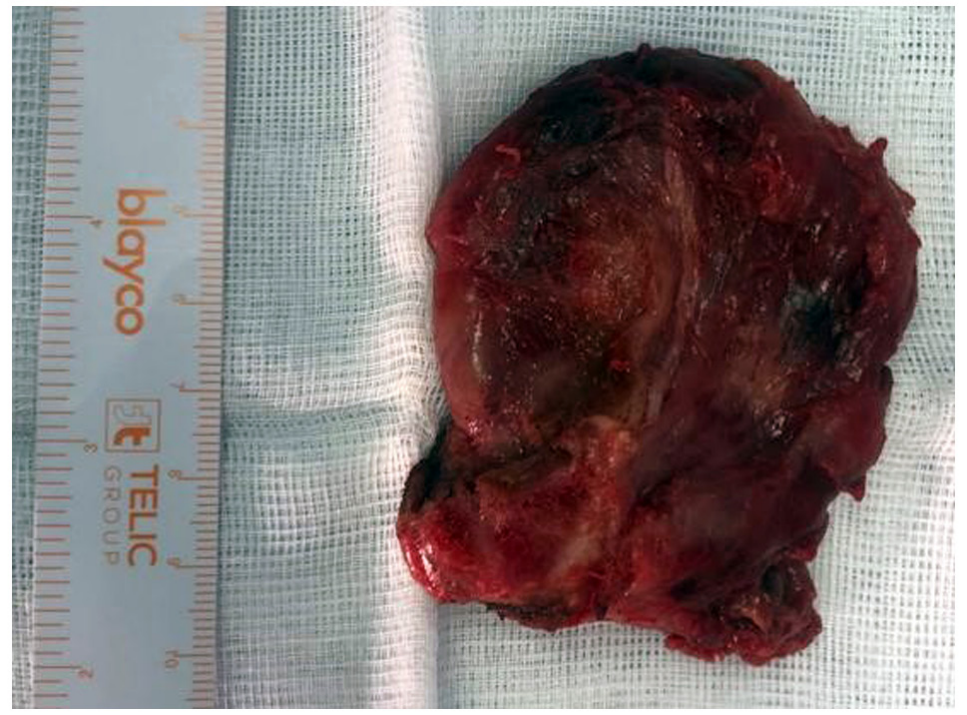
A right hemithyroidectomy was finally performed in the immediate surgery performed following diagnosis. A large abscess involving the entire right thyroid compartment was observed and drained carefully by cavity irrigation and the placement of a drain in the surgical bed.

After the diagnosis was confirmed, the patient was commenced on empirical intravenous antibiotics and an immediate surgery was indicated. Under general anesthesia, following orotracheal intubation with a video laryngoscope, a median cervicotomy was performed. A large abscess involving the entire right thyroid compartment was observed. Cultures were obtained, and finally, a right hemithyroidectomy was performed (Fig. 3), followed by extensive cavity irrigation and the placement of a drain in the surgical bed.

The immediate post-operative period required admission to the intensive care unit due to difficult airway management, and extubation was successfully performed. Oral tolerance was delayed by 1 week due to a positive dysphagia test, which was later repeated without abnormalities. Subsequently, he was initiated an oral diet without any incidents.

Cultures demonstrated the presence of *Escherichia coli*. He presented with fever spikes with negative blood cultures for microorganisms and with a good response to antibiotic therapy (gentamicin and ceftriaxone). Following 22 days in hospital and a CT scan indicating the radiological improvement of previously described cervical inflammatory changes, he was discharged from the hospital and he recovered uneventfully without any voice alterations, hypocalcemia or recurrence.

The results of the pathological analysis revealed chronic and acute inflammation, and areas of necrosis, along with thyroid tissue exhibiting no evidence of malignancy. Following histochemical analysis (PAS, Warthin-Starry and Ziehl) performed by the Pathological Anatomy Service of Príncipe de Asturias Teaching Hospital, microorganisms were not identified (data not shown).

## Discussion

Thyroid abscesses are a rare entity, with an incidence of <1% among thyroid gland pathologies and representing only 0.1% of surgical thyroid pathologies (4). It is an unusual condition due to the anatomical and physiological characteristics of the gland, which provides it with relative immunity (2). Some of the most common theories for this immunity include: The presence of both iodine and hydrogen peroxide in parenchymal tissue, which inhibits microbial growth; its extensive vascular supply and lymphatic drainage, which further protect it from infection; and furthermore, a fibrous thyroid capsule and fascial planes isolating it from other neck structures and shielding it from local spread of pathogens (5).

Thyroid abscesses have been observed to be more common among females than males (6), with a wide age range from 16 to 79 years (7), with the left side of the gland being more commonly involved. This left-sided predominance when the invasion of organisms is introduced through the blood supply is unexplained, but may possibly be related to the embryologist asymmetry of the fourth branchial arch, which forms the aortic and innominate arteries. The patient described herein was a 48-year-old male; he developed the lesion on the right side, despite the most conceivable origin of the abscess in this case being hematogenous spread.

In the adult population, thyroid abscesses typically affect patients with conditions, such compromised immune systems, or thyroid diseases such as Hashimoto's thyroiditis or thyroid cancer, while in the pediatric population, they are associated with local anatomical defects, such as pyriformis sinus fistula (8,9). In the present study, the patient described did not have any medical antecedents, and no anatomical abnormalities were identified in imaging diagnosis or in surgery; a pathological analysis also did not reveal thyroid diseases.

Hematogenous or lymphatic spread from a distal site of infection [usually following upper respiratory tract, pharynx, or middle-ear infections (2)] is considered to be a common cause of thyroid infection. In rare cases, they result from trauma or direct inoculation during invasive procedures, such as fine needle aspiration of the gland or central venous line placement (6). However, the exact infectious source or pathway is frequently unknown.

Generally, the flora that grows in microbiological cultures is polymicrobial, with *Staphylococcus aureus*, *Streptococcus pyogenes*, and Gram-negative microorganisms from the oropharynx being most commonly encountered (8,9). In the patient in the present study, it was considered that the cause of infection was a hematogenous spread from the acute prostatitis due to the finding of *Escherichia coli* in the cultures; this bacterium was also found in urine cultures taken the week before.

The classical presentation to thyroid abscess consists of fever, cervical pain and a painful mass (10). Associated signs are dyspnea, hoarseness, dysphonia and dysphagia (11). It is accompanied by leukocytosis, primarily due to polymorphonuclear cells. The diagnosis is confirmed through clinical findings and by diagnostic imaging (10): This includes an ultrasound and more specific computerized axial tomography.

The initial treatment (12) is based on empirical antibiotic therapy, with guided ultrasound-guided aspiration being recommended for culture to determine specific antibiotic treatment (13). In more severe cases, in patients with sepsis, thyroidectomy is the treatment of choice. For the patient described herein, an emergency surgery was performed due to the abnormalities in the laboratory test results and due to the restricted mobility of the neck and the ensuing risk of airway obstruction.

A hemithyroidectomy was finally performed in view of capillary hemorrhage from the capsule of abscess and resulting from the necessity of a biopsy. A neoplasm with an aggressive evolution can appear similar to an abscess, and it should be considered in the differential diagnosis when there are no identified predisposing factors for the abscess. A key factor is the presence of negative cultures obtained from the supposed pus collection.

Thyroid abscess is a severe and urgent condition. Certain complications can occur, such as the destruction of the thyroid or parathyroid glands, internal jugular vein thrombophlebitis, local or hematological spread to other organs, sepsis, and even abscess rupture or fistula formation into the esophagus or trachea (2).

In conclusion, although thyroid abscesses are an uncommon condition, they should be included when evaluating a thyroid mass, even in the absence of risk factors and particularly if it is an acute presentation with rapidly progressing symptoms. A thyroid ultrasound should be the first-line imaging modality. An early diagnosis and treatment with antibiotics and drainage are crucial to avoid severe morbidity.

## Data Availability

The datasets used and/or analyzed during the current study are available from the corresponding author on reasonable request.
